# Accelerating earth science discovery via multi-agent LLM systems

**DOI:** 10.3389/frai.2025.1674927

**Published:** 2025-11-12

**Authors:** Dmitrii Pantiukhin, Boris Shapkin, Ivan Kuznetsov, Antonia Anna Jost, Nikolay Koldunov

**Affiliations:** Alfred Wegener Institute for Polar and Marine Research, Bremerhaven, Germany

**Keywords:** multi-agent systems, large language models, geoscience data management, PANGAEA, retrieval-augmented generation, earth science informatics, scientific data discovery, autonomous AI agents

## Abstract

This Perspective explores the transformative potential of multi-agent systems (MAS) powered by Large Language Models (LLMs) in the geosciences. Users of geoscientific data repositories face challenges due to the complexity and diversity of data formats, inconsistent metadata practices, and a considerable number of unprocessed datasets. MAS possesses transformative potential for improving scientists’ interaction with geoscientific data by enabling intelligent data processing, natural language interfaces, and collaborative problem-solving capabilities. We illustrate this approach with “PANGAEA GPT,” a specialized MAS pipeline integrated with the diverse PANGAEA database for Earth & Environmental Science, demonstrating how MAS-driven workflows can effectively manage complex datasets and accelerate scientific discovery. We discuss how MAS can address current data challenges in geosciences, highlight advancements in other scientific fields, and propose future directions for integrating MAS into geoscientific data processing pipelines. In this Perspective, we show how MAS can fundamentally improve data accessibility, promote cross-disciplinary collaboration, and accelerate geoscientific discoveries.

## Introduction

Geoscience data archives, which serve as curated digital infrastructures for the systematic storage and distribution of Earth and environmental datasets, have grown to enormous scales, with large repositories such as PANGAEA, NASA’s Earth Observing System Data and Information System (EOSDIS), NOAA’s National Centers for Environmental Information (NCEI), and the Copernicus Climate Data Store (C3S) collectively hosting millions of heterogeneous datasets and several petabytes of data (Kobler et al., 1995; Felden et al., 2023). For example, PANGAEA alone contains more than 400,000 datasets derived from a variety of observational platforms ranging from shipboard expeditions and sediment cores to global climate model outputs (Felden et al., 2023). Despite this scale, data citation remains low, with over 93% of datasets being uncited (Robinson-García et al., 2016). At the core of this issue with data reuse are inadequate metadata standards, non-uniform data formats, and incomplete documentation (Gil et al., 2016). As a result, countless datasets remain underutilized or completely absent from scientific publications, resulting in missed opportunities for scientific discovery.

These challenges of managing and utilizing complex, heterogeneous datasets extend far beyond geosciences, impacting a wide array of scientific disciplines, where similar issues with data formats and metadata persist (Zhang and Zhao, 2015; Schnase et al., 2016; Pal et al., 2020). Large Language Models (LLMs), with their ability to parse unstructured data and reason across domains, provide a robust foundation for addressing these challenges (Bauer et al., 2024). LLMs have become powerful tools for information retrieval and analysis in various scientific fields (Guo et al., 2024). Recent Generative Pre-trained Transformer (GPT) models use advanced techniques like reinforcement learning and chain-of-thought reasoning (Wei et al., 2022). They excel at complex scientific problems, even surpassing PhD-level experts on tough benchmarks (Rein et al., 2023; Guo et al., 2025). These models can now perform multi-step reasoning, breaking down complex problems into manageable subtasks and synthesizing information from vast knowledge bases. Moreover, they can operate with tools and execute commands, expanding their problem-solving capabilities (Paranjape et al., 2023). These advanced capabilities suggest that LLM-driven approaches hold tremendous promise for geoscience data management.

A further evolution of LLM is expected in an agent-based mode, where models function as autonomous agents capable of performing tasks without constant human guidance, relying on tools, databases, internal memory and other resources (Guo et al., 2024). In this context, an agent is an LLM-based system that can perceive the environment, reason about the information it receives, and take actions to achieve specific goals. Such agents are often used collaboratively in a divide-and-conquer approach, deploying multiple specialized agents that can solve complex analytical problems as a group (Qian et al., 2023). This is particularly relevant in geosciences, where the diversity of data formats and the need for domain-specific expertise are significant challenges. By working together, these agents can efficiently handle heterogeneous datasets, bridge gaps between different branches of geoscience, and provide researchers with more holistic insights.

Single-agent and chat-completion approaches have already shown practical benefits in geosciences. Retrieval-augmented techniques (RAG; Lewis et al., 2020) which enable language models to dynamically access and incorporate information from external knowledge bases, have particularly enhanced domain-specific accuracy in climate science. For example, RAG approaches with curated climate reports have improved domain-specific QA tasks derived from Intergovernmental Panel on Climate Change (IPCC) documents (Vaghefi et al., 2023). Similarly, efforts such as “ClimSight” now provide climate projection information to non-specialist users by integrating LLMs with climate report data and model runs (Koldunov and Jung, 2024; Kuznetsov et al., 2025). Recent advances in RAG techniques have moved beyond simple document retrieval, incorporating multi-level retrieval mechanisms and knowledge graphs to enhance contextual understanding (Edge et al., 2024). These developments in retrieval-based systems are rapidly evolving and promise more sophisticated and accurate interactions with domain-specific knowledge bases.

Furthermore, several groups have explored ways to adapt general-purpose LLMs to the geosciences by further training them on large domains of geoscientific data. Notable work includes K2 (Deng et al., 2024) and GEOGALACTICA (Lin et al., 2023), which introduced new benchmarks and datasets for geoscience-specific tuning, as well as OceanGPT (Bi et al., 2023), which targets oceanographic tasks, and ClimateGPT (Thulke et al., 2024), which is fine-tuned on climate-related data.

Tool integration is a central technical feature of LLM based agents (Guo et al., 2024). Instead of relying solely on an LLM’s internal weights, LLM agents leverage tool wrappers, dynamic function calls, and API endpoints to execute domain-specific operations (Gim et al., 2024). In the geosciences, this integration has enabled the creation of specialized systems tailored to complex data and analysis needs. For example, Chen et al. proposed “GeoAgent,” a specialized LLM-based framework for geospatial data analysis that integrates a code interpreter, static analysis, and RAG (Chen et al., 2024). Another common use of single-agent systems with tool integration is in the application of search capabilities (Sun et al., 2023). For geosciences, an illustrative example is LLM-Find (Ning et al., 2025), which focuses on geospatial data extraction by providing LLMs with iterative debugging capabilities to retrieve spatial datasets (e.g., OpenStreetMap, weather APIs).

Although these projects have advanced LLM fine-tuning and the use of RAG and tools for geoscience challenges, no multi-agent frameworks have yet been developed that are tightly integrated with underlying geoscience databases.

## Emergence of the MAS

The transition from single-agent systems to multi-agent systems (MAS) is driven by the inherent complexity and heterogeneity of scientific workflows (Guo et al., 2024), which is particularly characteristic of the field of geosciences. While single-agent approaches can handle specific, well-defined tasks, they often struggle with interdisciplinary queries that require specialized knowledge across diverse data formats (e.g., NetCDF, CSV, seismic data) and domains. A monolithic agent architecture can become brittle when attempting to incorporate the breadth of tools and domain knowledge necessary (Guo et al., 2024).

Attempting to manage the vast array of required tools within a single agent often leads to “tool overload,” which increases the computational cost of token usage and decreases accuracy, as the model struggles with complex decision-making, potentially increasing hallucinations or tool misuse (Shen, 2024). MAS architectures justify their added complexity by offering specialization, modularity, and robustness. By deploying specialized agents, each equipped with domain-specific tools and knowledge bases, MAS can mimic the collaborative dynamics of human research teams (Qian et al., 2023), allowing for a divide-and-conquer approach to complex data challenges, which is essential for managing the diversity found in repositories like PANGAEA.

In such systems, each agent operates under predefined sets of instructions, and is equipped with domain-specific reasoning modules, customized knowledge databases, and direct interfaces to external tools and computational sandboxes (Guo et al., 2024). Advanced coordination strategies, frequently organized in hierarchical or graph-based flows, enable these agents to exchange intermediate results, negotiate optimal workflows, and iteratively refine partial outputs through chain-of-thought reasoning and reflection (Agashe et al., 2024; Pan et al., 2024). While MAS have shown impressive results in other domains, including collaborative code-generation in software engineering (Qian et al., 2023; Hong et al., 2023), coordinated planning in multi-robot systems (Mandi et al., 2023), modeling of complex societal interactions (Park et al., 2023), and strategic reasoning in game simulation (Wang et al., 2023), no integrated MAS solution has yet been applied to geoscientific data archives.

Nevertheless, initial MAS prototypes for geoscience-related tasks have begun to emerge. For example, ShapefileGPT (Lin et al., 2024) demonstrated a two-agent LLM framework for automating GIS shapefile processing, where a planner agent delegates spatial subtasks to a worker agent via a specialized function library. Another project, GeoLLM-Squad (Lee et al., 2025), introduced a multi-agent paradigm to remote sensing workflows by separating an orchestration agent from multiple domain-specific sub-agents, using open-source frameworks such as AutoGen to integrate modular API toolchains, interactive map UIs, intent-based tool selection, and workflow storage.

In practice, MAS architectures grounded in LLMs can span a wide spectrum of organizational structures, ranging from a single coordinating supervisor to fully autonomous “swarm” networks that collaborate without centralized control (Guo et al., 2024). Centralized systems rely on a top-level planner or “supervisor” agent (Qian et al., 2023) that breaks down tasks, delegates them to specialized sub-agents (e.g., retrieval, analytical, data transformation, validation), and then synthesizes final outputs. This approach, exemplified by hierarchical frameworks such as HuggingGPT (Shen et al., 2024), ensures a clear command-and-control mechanism, simplifies quality checks, and promotes consistent workflow management.

By contrast, decentralized models draw inspiration from social systems and swarm intelligence, letting each LLM agent operate more independently with local memory and goals, leading to emergent behaviors and robust parallelization (Huang et al., 2024). Hybrid approaches combine both strategies—for instance, dynamic orchestration via a transient “lead” agent while other agents freely negotiate tasks or refine each other’s outputs, mirroring human team dynamics (Huang et al., 2024).

One of the advantages of MAS is the ability for multiple agents to simultaneously use specialized tools, each solving different components of a complex problem. Examples might include invoking geospatial libraries such as Geospatial Data Abstraction Library (GDAL) for coordinate transformation, using NetCDF (Rew and Davis, 1990) or xarray (Hoyer and Hamman, 2017) to parse and aggregate spatiotemporal data cubes, and running specialized Python or R scripts for statistical analysis. Agents can perform iterative refinement steps (Madaan et al., 2024), re-checking results against data integrity constraints, filtering outliers using robust statistical thresholds, or querying uncertainty quantification modules that assess the credibility of results. This tool ecosystem allows MAS to move beyond static text generation, facilitating a closed-loop interaction model where data retrieval, pre-processing, quality control, analysis, and visualization occur under the guidance of autonomous, domain-aware agents. Reflection and self-critique loops can be implemented by designating a “validator” agent that routinely inspects outputs for internal consistency, methodological rigor, and adherence to community standards. Such approaches use iterative improvement pipelines that break down instructions into smaller steps, critique initial results, and apply further improvements (Ferraz et al., 2024).

The agent ecosystem is heterogeneous and includes various specialized agents-such as retrieval agents, analytical agents, data conversion agents, and reporting agents-that work together to accomplish various data management tasks (Guo et al., 2024). Retrieval agents incorporate retrieval-augmented generation (RAG) techniques, coupling embeddings from domain-specialized language models with vector databases that index geoscientific literature, vocabularies, and reference datasets (Lewis et al., 2020). Analytical agents may run topological anomaly detection on bathymetric grids, apply wavelet transforms to paleoclimate proxies, or compute ensemble mean biases in Coupled Model Intercomparison Project (CMIP)—class climate model runs (Eyring et al., 2016). Transformation agents handle unit conversions, project datasets onto common spatial grids, or standardize attribute names. Reporting agents synthesize results into structured outputs, annotate data lineage, and cite relevant publications. RAG-based knowledge infrastructures leverage curated metadata schema and persistent semantic stores that retain cross-session memory, allowing MAS to gradually refine a hypothesis or revisit previously unexplained anomalies. Iterative reasoning loops that incorporate domain feedback can detect subtle teleconnections in ocean–atmosphere systems, illuminate previously unrecognized correlations in coastal sedimentary records, or integrate high-resolution satellite measurements with legacy chemical tracers to map the evolution of marine biogeochemical cycles.

In the context of applying MAS to geoscience tasks, MAS can mimic the dynamics of interdisciplinary research teams, where specialists contribute their expertise, as has been done in software engineering (Qian et al., 2023). This synergy is essential for tackling challenges in Earth sciences, from predicting the response of ocean circulation to future warming scenarios to detecting subtle geologic signals of hazard precursors in tectonically active regions. The integration of specialized agents, robust tool usage layers, RAG-based semantic indexing, and adaptive architectural principles would establish MAS as advanced computational platforms for geoscientific discoveries.

## PANGAEA GPT: MAS architecture for geoscientific data discovery

**Figure 1:**
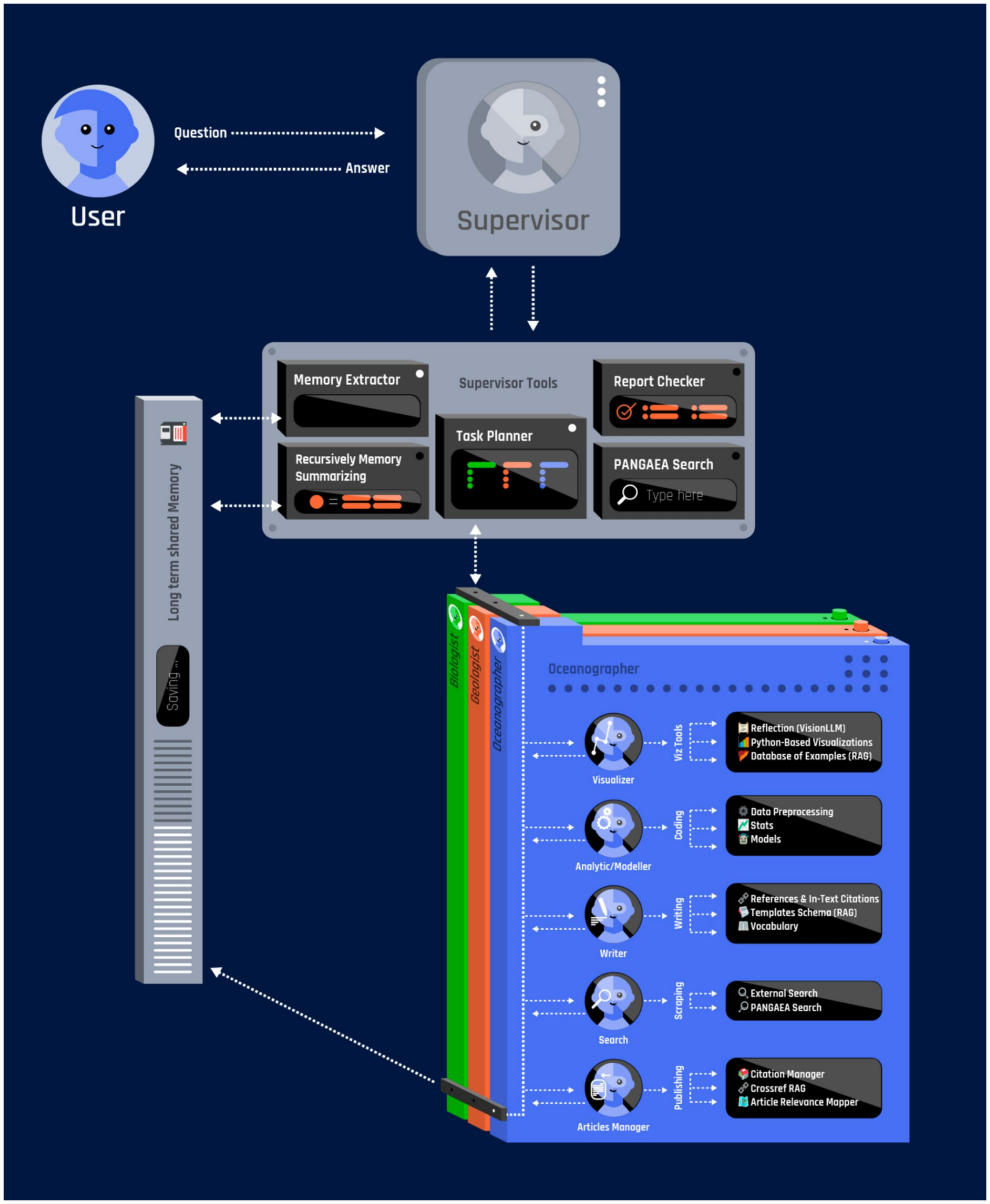
Conceptual framework multi-agent system (MAS) for geo-scientific data discovery.

To illustrate how the guiding principles described earlier can be put into practice, we propose a multi-agent system (MAS) architecture specifically designed for geoscience data management—focusing on large and diverse repositories such as PANGAEA (Felden et al., 2023). Based on our experience developing PANGAEA GPT—an open-source, LLM-driven multi-agent framework built upon the LangChain and LangGraph libraries (publicly available at github.com/CliDyn/pangaeaGPT, with a demo video at 10.5281/zenodo.15399454 and testable at huggingface.co/spaces/CliDyn/pangaeagpt) we illustrate how a centralized orchestration approach, where a supervisor agent directs domain-specific sub-agents (e.g., in oceanography, biology and geology), can be effectively implemented in geoscience contexts. This modular architecture allows the supervisor to spawn sub-agents on demand, adapting the system’s capabilities to the unique demands of each query. By referencing PANGAEA as a prime example of a heterogeneous database with unconventional formats and challenging metadata, we demonstrate how such a system can handle complex data workflows and provide robust reporting (Figure 1).

We chose a centralized orchestration approach, where the supervisor agent serves as the command-and-control node for the entire pipeline, handling sub-task delegation, resource allocation, and consolidation of final results among specialized agents (Figure 1). The system supports the dynamic creation of specialized agents based on the tasks assigned to it. Upon receiving user requests, the supervisor agent constructs agent subgraphs tailored to specific subdomains—oceanography, geology, climatology, ecology, or others—depending on the nature of the query. Each agent operates with localized memory buffers for context retention, set of tools and retrieval-augmented generation (RAG) capabilities that draw upon curated knowledge sources. Such a system is designed to efficiently search through diverse data collections, perform contextual analyses, produce high-quality visualizations, and ultimately generate comprehensive documentation.

In addition to assigning tasks, the supervisor agent handles memory and manages information flow across the system (Figure 1). Based on our experience running PANGAEA GPT, a multi-tier memory approach (storing short-term data in active memory and long-term data in a searchable database) was particularly effective for long-running sessions (Liu N. et al., 2024; Liu A. et al., 2024). Each agent runs locally and, after finishing its cycle, sends outputs back to the supervisor. To avoid overloading, the supervisor monitors resource usage, summarizes logs into short blocks, and then moves them into the long-term RAG database. Short-term context remains in the model’s direct context window, while extended data or partial results are stored in a vector database, retrievable on demand. This setup—short-term context paired with a stable long-term store-supports multi-step exploration without sacrificing critical details, and lowers computational costs during elaborate sessions.

### Ensuring reliability and scientific accuracy

In scientific contexts, the propensity of LLMs to hallucinate or propagate misinformation poses a significant risk (Kalai et al., 2025; Huang et al., 2025). PANGAEA GPT employs a multi-layered strategy to ensure reliability and compensate for the lack of deep, inherent geoscience knowledge in foundational LLMs. The core principle is Tool-Augmented Generation. The system is designed such that agents act primarily as orchestrators of deterministic tools rather than generators of scientific data or novel interpretations.

A key foundation of this architecture is the mandatory use of external tools to address domain-specific analysis needs (Figure 1). Upon deployment, each agent is provided with a dedicated “sandbox” containing domain-specific software, pre-installed packages, and necessary ancillary data (e.g., bathymetry, seafloor topography maps, multispectral satellite imagery, ocean color data, paleoclimate proxy records, atmospheric reanalysis fields). Analytical agents must use established libraries (e.g., xarray, GDAL, pandas) within these sandboxes to parse and analyze the actual data retrieved from repositories. The code executed by the agents is fully transparent and re-runnable by the user, ensuring verifiable results. This ensures that results are derived from the datasets and established scientific methods, rather than fabricated by the LLM.

Furthermore, the extensive use of Retrieval-Augmented Generation (RAG) grounds the agents’ reasoning in factual information (Lewis et al., 2020). Agents do not rely solely on the internalized knowledge of the LLM. The agent’s operational environment includes a RAG-accessible repository of domain-specific literature, sample visualizations, statistical analyses, and validated workflows. These features enhance the agent’s accuracy in answering user queries, reduce hallucinations by providing reliable domain references and best practice processing pipelines, and shorten reflection cycles by enabling rapid retrieval of reliable examples.

To further enhance accuracy, we implement specialized Reflection and Validation Modules (Shinn et al., 2023). In our PANGAEA GPT implementation, agents critically evaluate their outputs by invoking these modules. This includes statistical validation and the use of Visual Question Answering (VQA) modules to inspect visualizations. For instance, they can confirm whether unit scales match geoscientific norms or use VQA to ensure that depth axes are correctly reversed in oceanographic plots. By flagging suspicious metadata entries or unusual variable usage, these agents act as quality-control gates at each major step (data retrieval, analysis, and visualization), guiding the agent through iterative refinements until the final outputs meet the required quality standards.

## Limitations and challenges

While the MAS approach offers significant potential, it is crucial to acknowledge the current limitations and challenges associated with deploying these systems in geoscientific research.

The PANGAEA GPT framework, as presented in this Perspective, is a proof-of-concept. It currently lacks rigorous, quantitative empirical validation comparing its performance (e.g., success rates, efficiency) against traditional data discovery methods. A major challenge we encountered while deploying PANGAEA GPT, and a significant hurdle for the field generally, is verifying the correctness and relevance of multi-agent LLM outputs in the face of highly varied geoscientific data. Unlike software engineering, which typically uses standardized test suites or automated Quality Assurance (QA) workflows (Jimenez et al., 2023), Earth science has only a few domain-specific benchmarks that accommodate specialized terminologies and heterogeneous data (Bi et al., 2023; Zhang et al., 2024). This gap necessitates a human-in-the-loop evaluation framework (LangChain, Inc, 2025), where domain experts provide the crucial validation that automated benchmarks cannot yet offer. Ultimately, the goal of these systems is not to achieve full automation, but rather to serve as powerful assistants that accelerate discovery by augmenting expert judgment.

Another significant issue is the lack of any “imaging benchmark” that covers the range of visualization practices. This is further complicated by the fact that different programming languages are commonly used; ecologists or biologists often rely on R for plots (Gao et al., 2025), while oceanographers tend to prefer Python or Matlab. This diversity translates into an equally broad spectrum of plot types, from distribution maps and cross-sectional charts to correlation matrices, each governed by domain-specific conventions that generic validators rarely catch. The diversity of data types and visualization practices across geosciences complicates the development of universal validation metrics. A thorough evaluation constitutes a substantial research effort and is the focus of a planned future study. While a universal benchmark for LLM validation remains an important goal, our work indicates that domain-focused modules, like those implemented in PANGAEA GPT, are essential, particularly for detailed imaging tasks.

The implementation of MAS also introduces significant computational overhead. Running multiple LLM agents concurrently is computationally expensive, particularly when using high-parameter commercial models. This may present accessibility barriers for researchers or institutions with limited resources. While this cost must be weighed against the significant benefit of reduced “time-to-science” (accelerating data discovery and initial analysis), the trade-off remains a key consideration. Architectural optimizations, such as the multi-tier memory approach used in PANGAEA GPT (Liu N. et al., 2024; Liu A. et al., 2024), help manage token usage, but future work must explore the use of smaller, specialized LLMs to reduce the operational footprint. Furthermore, the rapid advancement of high-capability open-source models (Bai et al., 2023; Liu N. et al., 2024; Liu A. et al., 2024) offers a cost-effective alternative, allowing these systems to be deployed on local or institutional hardware, significantly reducing the operational footprint.

## Discussion and outlook

Looking forward, the integration of MAS systems into geoscientific research opens up entirely new opportunities for revitalizing previously underutilized historical data as well as more recently generated geoscientific data sources. Autonomous agent networks could systematically explore large repositories, identifying and summarizing historical datasets that have remained underutilized. By combining database-search agents with domain-specific expert analytic agents, this approach can help re-explore entire historical databases and interpret understudied collections. Ultimately, such a system may facilitate renewed engagement with valuable historical data and potentially give rise to new discoveries.

Another potentially promising direction is the use of MAS systems to assist in expedition planning. In the domain of shipping and commercial sectors, LLMs are already being proposed for planning to optimize routes and enhance safety (Pei et al., 2024). A potential MAS structure could be envisioned in which one agent first requests historical expedition data (taken directly from PANGAEA or other repositories), another agent checks current satellite products and climate forecasts, and yet another agent integrates predicted weather conditions and ocean currents. Together, these specialized agents would generate individualized expedition plans that optimize time spent at stations, for example, by coordinating dive schedules and sampling activities based on dynamic environmental factors. Such adaptability can streamline logistics and mitigate risks, particularly in remote or high-latitude regions prone to rapid weather changes, ultimately improving both the cost-effectiveness and safety of scientific expeditions. Additionally, the increasing use of Autonomous Underwater Vehicles (AUVs) in modern observatories (Wynn et al., 2014) makes MAS particularly attractive for operating such fleets during expeditions. These systems could control the AUVs, manage real-time data collection and transmission to repositories like PANGAEA, and use the analyzed data to dynamically re-optimize the AUVs’ routes, thereby enhancing the overall efficiency and adaptability of scientific missions.

A more radical idea would be to create a self-sufficient structure of autonomous, wandering chains of agents. One of the most far-reaching goals for MAS in geosciences is the formation of “wandering,” self-organizing systems of agents that continuously explore repositories, generating and testing new hypotheses without direct human guidance. These autonomous agent networks could continuously explore the scientific data landscape within repositories, suggesting new directions for research. Relying on unsupervised anomaly detection routines, they would be able, for example, to detect unexpected signals in real-time global seismic data or satellite ocean color imagery, thereby revealing potential new phenomena or hazard precursors. At the same time, a supervisor agent can spawn subordinate agents to propose mechanistic explanations for each anomaly, linking them to known processes. If the system would find plausible but unconfirmed signals, such as a region of unusual phytoplankton bloom, it could trigger additional analyses or domain-expert agents to investigate further, eventually communicating the summarized results to human scientists for more in-depth validation. Over time, this feedback loop could significantly shorten the time between discovery and research action, accelerating environmental insights that might otherwise remain buried in massive data stores. Such a self-governing swarm of agents could directly serve both experts and the general public, democratizing access to research data and broadening the scope of communication.

## Data Availability

The original contributions presented in the study are included in the article/Supplementary material, further inquiries can be directed to the corresponding author.
